# IEF-CSNET: Information Enhancement and Fusion Network for Compressed Sensing Reconstruction

**DOI:** 10.3390/s23041886

**Published:** 2023-02-08

**Authors:** Ziqun Zhou, Fengyin Liu, Haibin Shen

**Affiliations:** College of Information Science & Electronic Engineering, Zhejiang University, Hangzhou 310058, China

**Keywords:** compressed sensing reconstruction, strengthen error information supplement, greedy iterative, compressed domain fusion

## Abstract

The rapidly growing requirement for data has put forward Compressed Sensing (CS) to realize low-ratio sampling and to reconstruct complete signals. With the intensive development of Deep Neural Network (DNN) methods, performance in image reconstruction from CS measurements is constantly increasing. Currently, many network structures pay less attention to the relevance of before- and after-stage results and fail to make full use of relevant information in the compressed domain to achieve interblock information fusion and a great receptive field. Additionally, due to multiple resamplings and several forced compressions of information flow, information loss and network structure redundancy inevitably result. Therefore, an Information Enhancement and Fusion Network for CS reconstruction (IEF-CSNET) is proposed in this work, and a Compressed Information Extension (CIE) module is designed to fuse the compressed information in the compressed domain and greatly expand the receptive field. The Error Comprehensive Consideration Enhancement (ECCE) module enhances the error image by incorporating the previous recovered error so that the interlink among the iterations can be utilized for better recovery. In addition, an Iterative Information Flow Enhancement (IIFE) module is further proposed to complete the progressive recovery with loss-less information transmission during the iteration. In summary, the proposed method achieves the best effect, exhibits high robustness at this stage, with the peak signal-to-noise ratio (PSNR) improved by 0.59 dB on average under all test sets and sampling rates, and presents a greatly improved speed compared with the best algorithm.

## 1. Introduction

At present, the acquisition, transmission, and processing of information are proliferating, which brings great challenges to data storage and transmission. Meanwhile, the amount of sampled data is greatly restricted due to limitations in power, bandwidth, or sampling time in some extreme cases. Fortunately, the Compressed Sensing (CS) [1] theory has partially broken through the limitations of the traditional Nyquist sampling theory [2]. A reduced sampling rate can achieve low-cost and efficient data compression and is beneficial for decreasing the burden of storage and transmission.

In recent years, a tremendous number of algorithms have been proposed to address CS reconstruction, including two main categories: traditional methods and Deep Learning (DL) methods.

Traditional methods are usually based on theoretical guarantees to solve undetermined equations and obtain optimal results through gradual iteration, so they inevitably suffer from the high computational cost dictated by interactive calculations [3] and are hard to execute by parallel computing.

Different from traditional methods, DL methods have been applied to solve image CS reconstruction problems. They can map from compressed data to original signals by training a larger number of parameters in Deep Neural Networks (DNNs) with superior inference time and reconstruction quality. Of course, they are subject to some defects.

First, few methods, such as AMPNet [4], can make ultimate use of information in the compressed domain. However, the current region of compressed information will be applied to reconstruct the corresponding image blocks only, leading to a small receptive field [3]. Second, the intermediate features of previous iterations are completely ignored during reconstruction, although they can guide the recovery of the following iterations effectively. Last but not least, most relevant works inevitably suffer from a large amount of information loss caused by resampling and channel compression. The intermediate results should be compressed and resampled to obtain error information for supplementing the iterative reconstruction.

In view of the previous studies and limitations mentioned above, an IEF-CSNET is proposed here. The main contributions of this work are listed as follows:

- Based on the Compressed Information Extension (CIE) module, information in the compressed domain is fully utilized for high-dimensional fusion, greatly expanding the receptive field of DNN methods.
- In consideration of the initial image and the error enhancement image recovered by previous iterations, the Error Comprehensive Consideration Enhancement (ECCE) module can incorporate the enhancement information into the output flow more efficiently.
- To solve the information compression due to obtaining errors, an Iterative Information Flow Enhancement (IIFE) module is proposed to complete iterative and progressive recovery during loss-less information transmission.
- Combined with CIE, ECCE, and IIFE, the IEF-CSNET is proposed. On this basis, several experiments and visual analyses of its effectiveness are performed. Under all test sets and sampling rates, the average increase is approximately 0.59 dB, and the operating speed is improved by orders of magnitude from the state-of-the-art (SOTA) method.

The rest of this paper is organized as follows. In Section 2, the CS and some related works are introduced and analyzed. The proposed IEF-CSNET is elaborated on in Section 3. The settings and substantial results of the experiments are provided in Section 4. Finally, the conclusion is summarized in Section 5.

## 2. Related Works

In this section, a brief review of CS and some benchmark works are provided, which can be generally divided into traditional methods and DL methods.

### 2.1. Compressed Sensing and Traditional Methods

CS can sample and compress the signals simultaneously, breaking through the limitation of the Shannon Nyquist sampling theorem [1]. Mathematically, CS reconstruction aims to infer the original signal $X\in R^{N\times 1}$ from the CS measurements $Y\in R^{M\times 1}$.  Measurement Y can be obtained by a nonadaptive linear projection $Y=\Phi X,\Phi \in R^{M\times N}$, whereby the CS sampling ratio is defined as R=M/N. However, if there are sparse representations ΨX of X in domain Ψ, the typical ill-posed problems can be solved by measurement Y correctly with high probability because it can be transformed into the following expression, which is an optimization:

(1) $$\min_{X}{∥\Psi X∥}_{p},\;s.t.\;Y=\Phi X$$

where ${∥*∥}_{p}$ means the *p* norm of vector * to characterize the sparsity of the vector. Thanks to CS theory, the loads of data acquisition, transmission, and storage can be greatly reduced.

Many traditional works have been performed to design the sampling matrix [5] and reconstruct X from the CS measurement Y. Convex optimization methods achieve accurate and robust recovery results by translating the nonconvex constraint into a convex constraint [6]. Greedy algorithms, such as Matching Pursuit (MP) [7], Orthogonal MP (OMP) [8], and stagewise OMP [9], generally obtain results based on the iterative residual, achieving lower reconstruction quality but sharing low computational complexity [10]. Refs. [11,12,13,14] take the source at Projected Landweber [15] and reconstruct by successively projecting and thresholding. The Total Variation (TV) [16] establishes more sophisticated models and focuses more on image priors. Nevertheless, details could be lost due to the too-smooth constraint.

Although some traditional methods have been widely applied practically, such as wireless sensor network [17], hyperspectral imaging [18], Magnetic Resonance Imaging (MRI) [19,20], underwater sensors [21], and aerospace [22], they usually suffer from too slow a running speed, due to the inevitable large numbers of iterations and heavy matrix operations, which must be executed in a nonparallel way in each iteration. In addition, it is difficult to draw enough prior knowledge from big data, causing performance bottlenecks.

### 2.2. Deep Learning Methods

By exploiting massive paralleled Graphic Processing Unit (GPU) processing architectures and large amounts of training data, DL methods are mainly composed of various high-density parallel computing processes and can achieve superior reconstruction quality and computational complexity when compared to traditional methods. ReconNet  [23], the first DL method, presents a noniterative and extremely fast algorithm to reconstruct images from CS measurements. Block Compressed Sensing (BCS) is suitable for image sampling and compression in resource-constrained applications [24]. However, some methods are prone to suffering from blocking artifacts due to the independent recovery among blocks, so it is necessary to cascade a time-consuming filter, BM3D. Instead of the fixed sampling matrix, DR2-Net [25], CSNET [10], and CSNET+ [26] implement the learnable fully connected layer and convolution layers for sampling. They all improve the reconstruction quality by stacking several residual learning blocks. NLR-CSNET [27] and DPA-Net [28] introduce a 3D encoder/decoder and a dual-path network based on the attention mechanism for better reconstruction quality. The encoder and decoder in [27] with channel attention motivate the effective skip links. However, these straightforward methods are largely unprincipled black boxes that are difficult to train and often-times specific to a single measurement matrix [29].

Deep unfolding methods incorporate traditional iterative reconstruction and DNNs, and they can map each iteration into a series of network layers that preserve interpretability and performance. Learned Denoising-based Approximate Message Passing (LDAMP) [29] combines the Denoising-Based Approximate Message Passing (D-AMP) algorithm and neural network and runs over 50 times faster than BM3D-AMP. Inspired by the Iterative Shrinkage-Thresholding Algorithm (ISTA), ISTA-Net+ [30], and ISTA-Net++ [31] design deep networks,  all parameters are learned end-to-end rather than hand-crafted. The difference is that ISTA-Net++ uses a cross-block learnable sampling strategy and achieves multi-ratio sampling and reconstruction in one model but leads to a low robustness of all compression ratios. Moreover, OPINE-Net [32] is a variant of ISTA-Net+ and adopts end-to-end training and learning to explore adaptive sampling and reconstruction. An unfolded LSTM network is utilized in video compression sensing, deeply fusing the intra- and interframe spatial–temporal information [33]. Finally, AMPNet [4] unfolds the iterative denoising of the AMP algorithm and shows a better reconstruction accuracy with high reconstruction speed.

Generally, due to the memory limitation and compatibility with input size, the sampling matrix can not share the same size as the original image. Thus, almost all images are initially reconstructed based on blocks and then denoised, leading to insufficient information integration in the compressed domain and small receptive fields. Some valuable compressed information from the other adjacent blocks can be extracted to assist in the reconstruction of the current block. This is in line with the data-oriented training mechanism because DL methods are good at high-dimensional mapping and learning autonomous knowledge. The methods mentioned above employ the solution of iterative progressive reconstruction for better performance. In this case, the processed results of intermediate iterations are considered to contain a wealth of information. In the repeated process, the data reconstructed painstakingly will be forcibly compressed or discarded and are expected to be resampled to the same size as input Y to obtain the difference in the compressed domain. Meanwhile, it is inconsistent with the advantage of the parallel computing of DL methods. Moreover, the generous results underutilized may cause many network redundancies. In the meantime, it is frustrating that the results calculated in previous iterations are ignored in most methods, and the previous features are supposed to be better used to explore and analyze which modes of information are difficult to recover. In this way, they can effectively strengthen the subsequent reconstruction.

## 3. Methods

### 3.1. Overview of Proposed Method

**Figure 1:**
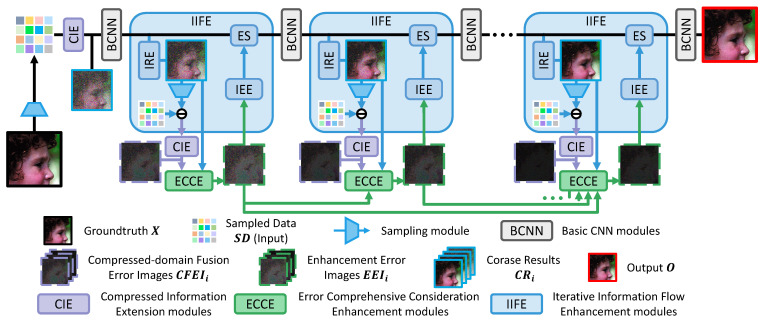
Overview of the proposed IEF-CSNET. The real image X to be sampled and the final output O are marked by solid black and red wireframes, respectively. X also plays the role of ground truth for training. (1) The CIE modules (displayed as purple blocks and introduced in Section 3.2) receive information in the compressed domain (Sampled Data (SD) or the error between SD and Stage Sampled Data (${\mathrm{SSD}}_{i}$)) as input. Then, CIE modules output the 1st iteration Corase Result (${\mathrm{CR}}_{1}$) or Compressed-domain Fusion Error Image ${\mathrm{CFEI}}_{i}$. (2) ECCE modules (displayed as green blocks and introduced in Section 3.3) achieve ${\mathrm{EEI}}_{i}$ by summarizing ${\mathrm{CR}}_{i}$, ${\mathrm{CFEI}}_{i}$, and ${\mathrm{EEI}}_{2∼(i-1)}$ from ECCE modules in previous iterations. (3) The $N_{I}$ IIFE modules (displayed as blue blocks) aim to reconstruct images more effectively stage by stage. Each IIFE module can be transmitted with little information loss. It is composed of the Sampling S(·), Iterative Result Extraction (IRE), Iterative Error Extension (IEE), and Error Supplement (ES) modules, which are all introduced in Section 3.4. (4) A total of 3 3 × 3 convolution layers with 32 channels and ReLU layers are employed as the Basic CNN (BCNN) module for nonlinear mapping and output the Intermediate Feature ${\mathrm{IF}}_{i}$.

This subsection introduces the overall structure and exhibits the whole prediction pipeline of the proposed method. Figure 1 shows how the submodules are closely combined with each other and organized into a more effective whole. The complete method is implemented by the following detailed submodules:

- The CIE module expands and integrates the information elements in the compressed domain to output ${\mathrm{CR}}_{1}$ and the Compressed-domain Fusion Error Image ${\mathrm{CFEI}}_{i}$, which can take greater advantage of the measurements in each iteration and achieve a larger receptive field (Section 3.2).
- The ECCE module outputs the Enhanced Error Image ${\mathrm{EEI}}_{i}$ by taking ${\mathrm{CR}}_{i}$, ${\mathrm{CFEI}}_{i}$, and ${\mathrm{EEI}}_{2∼(i-1)}$ of the previous iterations into consideration. In this way, the error and residual can be accurately predicted with high robustness to supplement the following reconstruction more efficiently (Section 3.3).
- Based on the IIFE module, the Intermediate Features ${\mathrm{IF}}_{i}$ and ${\mathrm{EEI}}_{i}$ can be supplemented progressively and fused more smoothly under loss-less information transmission while the sampling is repeated in the iterative reconstruction process (Section 3.4).

Meanwhile, pseudo code matching with the structure diagram (Figure 1) is provided as follows (Algorithm 1) to explain the inference more intuitively.
**Algorithm 1** Prediction of IEF-CSNET.1:PREDICT (Input: SD = Φ(X))2: **for** each $i\in [1,N_{I}]$ **do**3:  **if** i==1 **then**4:   ${\mathrm{CR}}_{1}$ = ${\mathrm{CIE}}_{1}$(SD)5:   ${\mathrm{IF}}_{1}$ = ${\mathrm{BCNN}}_{1}$(${\mathrm{CR}}_{1}$)6:  **else**7:   ${\mathrm{CR}}_{i}$ = ${\mathrm{IRE}}_{i}$(${\mathrm{IF}}_{i-1}$)8:   ${\mathrm{SSD}}_{i}$ = $S({\mathrm{CR}}_{i})$9:   ${\mathrm{CFEI}}_{i}$ = ${\mathrm{CIE}}_{i}$(${\mathrm{SSD}}_{i}$-SD)10:   ${\mathrm{EEI}}_{i}$ = ${\mathrm{ECCE}}_{i}$(${\mathrm{CR}}_{i}$,${\mathrm{EEI}}_{(i-1)∼2}$,${\mathrm{CFEI}}_{i}$)11:   ${\mathrm{IF}}_{i}^{\prime }$ = ${\mathrm{ES}}_{i}$( ${\mathrm{IF}}_{i-1}$, ${\mathrm{IEE}}_{i}$(${\mathrm{EEI}}_{i}$))12:   ${\mathrm{IF}}_{i}$ = ${\mathrm{BCNN}}_{i}$(${\mathrm{IF}}_{i}^{\prime }$)13:  **end if**14: **end for**15: **return** ${\mathrm{IF}}_{N_{I}}$

### 3.2. Compressed Information Extension (CIE)

**Figure 2:**
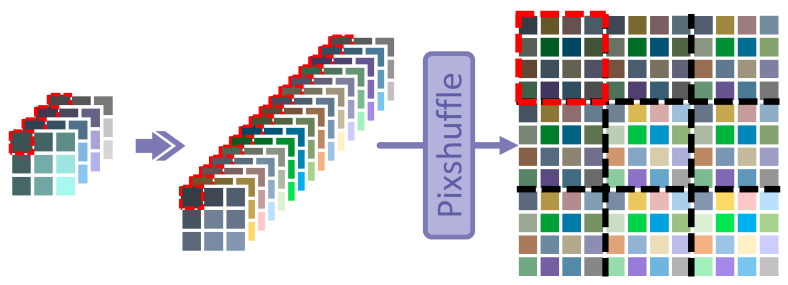
The base upsampling module in most related works. SD is displayed as a feature map with 4 channels to represent that each block is sampled into 4 measurements, and then it is upsampled and expanded into 16 channels, which is marked as the purple arrow. After that, the feature map is stretched into the same shape as the ground truth X by pixelshuffle [34]. The image is processed separately block by block, which is shown and divided by the black dotted line. For example, the SD marked by the red dotted line is just processed and stretched into the block in the upper left corner of the image by itself.

After analyzing and considering most of other related works, it is realized that the images are processed and divided into many blocks, which will be compressively sampled and independently reconstructed as the CR blocks. In this process, only measurements of the corresponding region block are employed for reconstruction, which are shown and represented by the red dotted boxes in Figure 2. Under these circumstances, the reconstruction of each block is competed independently. It can be summarized as follows:

(2) $${\mathrm{CR}}_{\left(i,j\right)}=PS\left(upsampling\left({\mathrm{SD}}_{\left(i,j\right)}\right)\right)$$

where ${\mathrm{SD}}_{\left(i,j\right)}$ and ${\mathrm{CR}}_{\left(i,j\right)}$ mean the sampled data and the coarse reconstruction of block (i,j), respectively. The 1×1 convolution or full connection layer is adopted for upsampling, and PS(·) means the Pixelshuffle option [34].

Some methods cannot prevent reconstruction from blocking artifacts under a low sampling rate, such as [23]. Because the number of measurements in ${\mathrm{SD}}_{\left(i,j\right)}$ is severely insufficient when extremely compressed, small disturbances of measurements from different blocks may cause overall offsets of each ${\mathrm{CR}}_{\left(i,j\right)}$ after the reconstruction. Thus, the helpful information in the compressed domain should be drawn from related blocks and fused into the current block to obtain more valuable guidance for reconstruction.

Meanwhile, it is found that only a few methods can achieve a relatively large receptive field while reconstructing. During upsampling, other methods [4,23,26,31,35] only focus on the corresponding measurements compressed from the target single block while reconstructing. Under normal conditions, a larger receptive field tends to bring better performance. For example, CSformer [3] integrates the characteristics of Transformer [36] to obtain a large receptive field in theory and obtain SOTA performance. However, an inference speed that is too low may be very fatal. It is even slower than some traditional algorithms, so the advantage of DL methods cannot be exhibited. Currently, GPU devices are good at parallel computing with high throughput, which makes the calculation of multichannel feature maps possible, promoting the feasibility of the methods introduced later.

**Figure 3:**
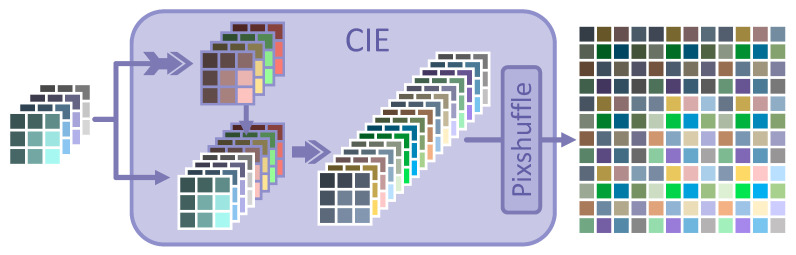
The pipeline of the CIE module. First, the fusion of measurements in the compressed domain is realized by 3×3 convolution (indicated by the purple arrow with tail) to obtain another Expanded Sampled Data (ESD), which is marked in a purple solid wireframe and is concatenated to the SD to maintain that the original measurements will not be averaged. Then, this fusion of SD and ESD, which is noted as EFSD, can be upsampled and reshaped in a way similar to that given in Figure 2.

For the reasons and analysis mentioned above, the CIE module is developed as a solution. It can take full advantage of measurements in the compressed domain and share a super large receptive field. In addition, it is suitable for GPU devices. To our knowledge, a similar design has not been used in relevant works at this stage. The pipeline is illustrated in Figure 3.

EFSD and ${\mathrm{CR}}_{1}$ at the 1st iteration can be calculated as follows:

(3) $$\begin{array}{cc} \mathrm{ESD} & =\mathrm{SD}*\Theta (W_{\left(3\times 3\right)},b)\\ \mathrm{EFSD} & =\left\{\mathrm{SD},\mathrm{ESD}\right\}\\ {\mathrm{CR}}_{1} & =PS\left(upsampling\left(\mathrm{EFSD}\right)\right) \end{array}$$

where (·)∗Θ(W,b) represents the convolution option through parameter groups W and b.

In the related works, each block with 32×32 pixels is compressed into 1×1 pixels with 1024×R channels in the compressed domain. Thus, the receptive field may be limited to the current block. However, the fusion of SD and ESD can easily achieve three or even more times the effect of the receptive field from other methods. The CIE modules can ensure the information perception for at least 32×3 receptive fields at each iteration. In addition, the checking, judging, and consulting of the surrounding blocks can be realized in advance at the initial reconstruction of ${\mathrm{CR}}_{1}$ for better reconstruction of the current block.

Similar to Equation (3), when the iteration i>=2, the ${\mathrm{CFEI}}_{i}$ can be calculated by the CIE module from ${\mathrm{SSD}}_{i}-\mathrm{SD}$, as follows:

(4) $$\begin{array}{cc} {\mathrm{ESD}}^{\prime } & =\left({\mathrm{SSD}}_{i}-\mathrm{SD}\right)*\Theta \left(W_{\left(3\times 3\right)},b\right)\\ {\mathrm{EFSD}}^{\prime } & =\left\{\left({\mathrm{SSD}}_{i}-\mathrm{SD}\right),{\mathrm{ESD}}^{\prime }\right\}\\ {\mathrm{CFEI}}_{i} & =PS\left(upsampling\left({\mathrm{EFSD}}^{\prime }\right)\right) \end{array}$$

where ${\mathrm{SSD}}_{i}-\mathrm{SD}$ is noted as the error between SD and ${\mathrm{SSD}}_{i}$. ${\mathrm{CFEI}}_{i}$ is the image of the residual error after more comprehensive consideration in the compression domain.

In this way, EFSD and ${\mathrm{EFSD}}^{\prime }$ can be obtained as more effective information in the compressed domain without damaging or impacting original measurements. In the meantime, CIE modules make larger receptive fields come true, tending to better reconstruction performance.

### 3.3. Error Comprehensive Consideration Enhancement (ECCE)

**Figure 4:**
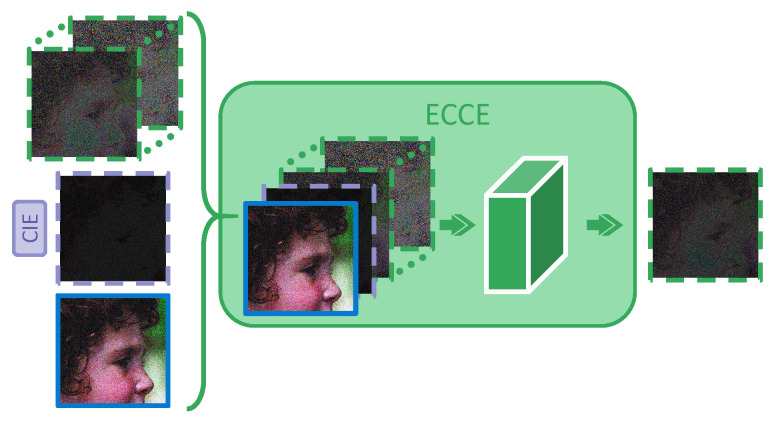
The pipeline of the ECCE module. In the iteration *i*, the module ${\mathrm{CIE}}_{i}$ and its output ${\mathrm{CFEI}}_{i}$ are both marked in purple. The ${\mathrm{CR}}_{i}$ and the previous EEIs output by ECCEs are marked as blue solid wireframes and green dotted boxes, respectively. ${\mathrm{ECCE}}_{i}$ takes ${\mathrm{CR}}_{i}$, ${\mathrm{CFEI}}_{i}$, and ${\mathrm{EEI}}_{\left(2∼(i-1)\right)}$ as input and outputs ${\mathrm{EEI}}_{i}$. The green arrows represent the 3×3 convolution and activation options. I/O channels of the two convolution layers are i/32 and 32/1, respectively, for nonlinear mapping and comprehensive enhancement of EEI.

Most related works fail to make full use of the previous iteration results and may ignore the connections during progressive reconstruction to some extent. First, the task in each iteration is consistent during the entire process, and the errors between ${\mathrm{CR}}_{i}$ and X can be predicted in each iteration. The residual error is gradually reduced during the iteration and shares the same target so that the previous residuals are valuable to guide the next stage of error prediction. Based on this, an ECCE module is proposed here to refine the reconstruction, the pipeline of which is shown in Figure 4.

The input of ${\mathrm{ECCE}}_{i}$ of iteration *i* can be achieved as follows:

(5) $$\begin{array}{cc} {\mathrm{Input}}_{i} & =\left\{\begin{array}{cc} \left\{{\mathrm{CR}}_{2},{\mathrm{CFEI}}_{2}\right\}, & i=2\\ \left\{{\mathrm{CR}}_{i},{\mathrm{EEI}}_{2∼(i-1)},{\mathrm{CFEI}}_{i}\right\}, & 2<i\leq N_{I} \end{array}\right. \end{array}$$

where ${\mathrm{CFEI}}_{i}$ represents the output of ${\mathrm{CIE}}_{i}$ (Equation (4)). ${\mathrm{CFEI}}_{i}$ and ${\mathrm{CR}}_{i}$ can be understood as the abstract summary and the prediction of target error in iteration *i*, respectively. The set of ${\mathrm{EEI}}_{2∼(i-1)}$ means that ${\mathrm{ECCE}}_{i}$ considers the previous results of iteration [2,i−1]. They are all employed as the input of ${\mathrm{ECCE}}_{i}$ to predict the ${\mathrm{EEI}}_{i}$. ${\mathrm{ECCE}}_{i}$ makes the fusion of multiple pieces of information more sufficient by coding the input as follows:

(6) $$\begin{array}{cc} {\mathrm{EEI}}_{i} & =CNN_{32-1}\left(CNN_{i-32}\left({\mathrm{Input}}_{i}\right)\right) \end{array}$$

where $CNN_{i-j}\left(\cdot \right)$ represents the 3×3 convolution option and one ReLU layer, with *i* input channels and *j* output channels.

The ECCE module has the following advantages. First, based on ${\mathrm{EEI}}_{2∼(i-1)}$ from previous stages, the proposed module predicts the ${\mathrm{EEI}}_{i}$ more accurately and realizes more adaptive reconstruction. Under the training of a large amount of data, the network can remember and even judge which information mode is difficult to reconstruct. Because of paying more attention to the connection of previous results, some components are always maintained throughout the whole process, and then the network will focus more on these stubborn questions to strengthen the final output. Second, different from other methods, ECCE receives the ${\mathrm{CR}}_{i}$ and ${\mathrm{CFEI}}_{i}$ as parts of the input at each iteration (as illustrated in Figure 4 and Equation (5)). In addition, it aims to combine the intermediate results with errors to analyze the targeted shortcomings in ${\mathrm{CR}}_{i}$ in the current situation to better integrate the errors later. Finally, two-layer CNN is employed for dimension expansion and compression to achieve a similar effect as the autoencoder for deeper information fusion.

### 3.4. Iterative Information Flow Enhancement Module (IIFE)

**Figure 5:**
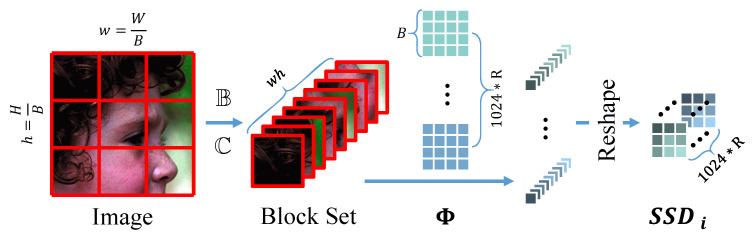
Parallel sampling module S(·). The dimension of the sampling matrix Φ is set as 32×32×(1024×R). The input image of S(·) is divided into blocks with 32×32 pixels, which is the same size as one channel of Φ. The dividing line is shown in red, where $w=\frac{W}{32}$ and $h=\frac{H}{32}$ represent the number of blocks in the width and height, respectively. The block set will be sampled as a 1×1×(w∗h) measurement for a total of 1024×R parallel executions. Therefore, the sampling rate can be understood as $\frac{1024\times R}{32*32}=R$.

The existing hardware system performs poorly in accelerating the large kernel convolution, so images or feature maps are no longer sampled by convolution options with a large kernel size. Instead, the sampling module is completed by multichannel parallel multiplication due to no overlap among different blocks during sampling. It is noted as S(·) and shown in Figure 5.

First, the image is divided into many blocks by B=32.

(7) $$\begin{array}{cc} I_{\left(i,j\right)} & =I[(i-1)*B:i*B,(j-1)*B:j*B]\\ I_{B} & =B\left(I\right)=\left\{\begin{array}{cccccccc} \; & I_{\left(1,1\right)}, & \; & I_{\left(1,2\right)}, & \; & \cdots & \; & I_{\left(1,w\right)}\\ \; & I_{\left(2,1\right)}, & \; & I_{\left(2,2\right)}, & \; & \cdots & \; & I_{\left(2,w\right)}\\ \; & \vdots & \; & \vdots & \; & \cdots & \; & \vdots \\ \; & I_{\left(h,1\right)}, & \; & I_{\left(h,2\right)}, & \; & \cdots & \; & I_{\left(h,w\right)} \end{array}\right\} \end{array}$$

where $w=\frac{W}{B}$ and $h=\frac{H}{B}$ represent the numbers of blocks in width and height, respectively. Then the blocks are concatenated as a whole feature map at dimension *C*.

(8) $$\begin{array}{cc} I_{C} & =C\left(I_{B}\right)=\left\{I_{\left(1,1\right)},\cdots I_{\left(h,w\right)}\right\} \end{array}$$

where C(·) represents the option of concatenating. In this way, $I_{C}\in R^{32*32*(wh)}$ becomes the set of blocks being sampled, and the ${\mathrm{SSD}}_{i}\in R^{w*h*(1024*R)}$ in iteration *i* can be achieved as follows:

(9) $$\begin{array}{cc} {\mathrm{SSD}}_{i} & =S\left({\mathrm{CR}}_{i}\right)=reshape\left(C\left(B\left({\mathrm{CR}}_{i}\right)\right)\cdot \Phi \right) \end{array}$$

where reshape(·) represents the reverse operation of B(·), which aims to organize $\left(C(B\left({\mathrm{CR}}_{i}\right))\cdot \Phi \right)\in R^{\left(w*h)*(1024*R\right)}$ into $R^{w*h*(1024*R)}$ (Figure 5). It is noted that S(·) should be executed repeatedly with the same sampling matrix Φ in each IIFE.

**Figure 6:**
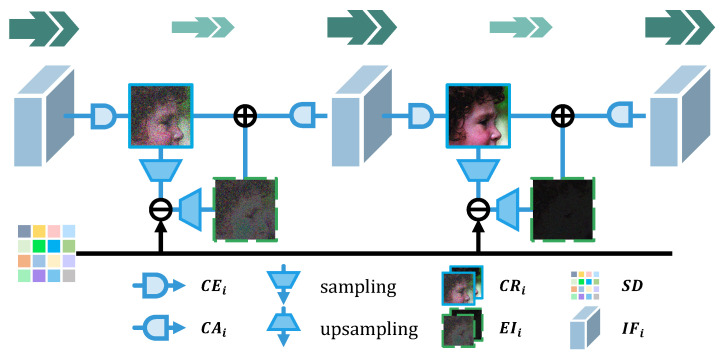
Base module in related works. ${\mathrm{CR}}_{i}$ and error images ${\mathrm{EI}}_{i}$ are represented as blue solid wireframes and green dotted wireframes, respectively. The ${\mathrm{IF}}_{i}$ is marked as blue cubes. The green double arrows mark the total number of feature maps, which can also be understood as the reconstructed information flow contained in the network.

In other related methods, ${\mathrm{IF}}_{i}$ is normally compressed into one-channel ${\mathrm{CR}}_{i}$ by $CE_{i}$ and sampled to achieve the error in the compressed domain, as shown in Figure 6. The residual error is upsampled into another error image ${\mathrm{EI}}_{i}$ with the same size as X. Then, ${\mathrm{EI}}_{i}$ is added to the main branch directly. Following that, the one-channel feature ${\mathrm{CR}}_{i}$ is augmented in the channel dimension to generate ${\mathrm{IF}}_{i+1}$ for the next step. In such a way, most intermediate results will be lost during the forced compression, which is indicated by the lighter and smaller green arrows in Figure 6. This is a large bottleneck of the network performance.

**Figure 7:**
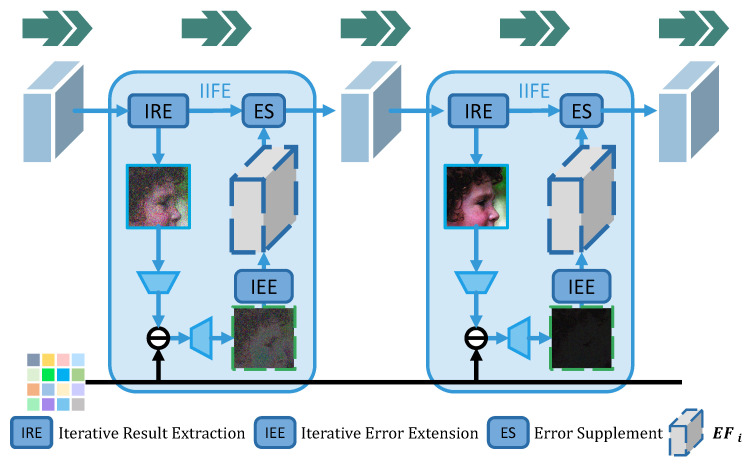
IIFE module. IRE, IEE, and ES modules, marked with dark blue boxes, aim to extract the ${\mathrm{CR}}_{i}$ from ${\mathrm{IF}}_{i}$, encode and extend the ${\mathrm{EI}}_{i}$ to output abundant information of Error Features ${\mathrm{EF}}_{i}$, and supplement the main branch ${\mathrm{IF}}_{i}$ by ${\mathrm{EF}}_{i}$, respectively. Additionally, ${\mathrm{IF}}_{i}$ and ${\mathrm{EF}}_{i}$ are represented as blue and gray cubes, respectively. Compared with Figure 6, there is no forced waste and compression of the information flow marked by green arrows, thus achieving a smoother reconstruction.

Within this work, the IIFE module is proposed to make full use of ${\mathrm{IF}}_{i}$, which is shown in Figure 7. Based on ${\mathrm{SSD}}_{i}-\mathrm{SD}$ in the compressed domain, a relatively complete ${\mathrm{EI}}_{i}$ can be predicted by upsampling. Meanwhile, the ${\mathrm{EI}}_{i}$ can be expanded to enrich information ${\mathrm{EF}}_{i}$ by the IEE and to adjust the main branch features in all channels. In this case, the fusion of ${\mathrm{EF}}_{i}$ and ${\mathrm{IF}}_{i}$ can be smoother without any information being lost. Therefore, ${\mathrm{IF}}_{i}$ with diverse information in different channels can be corrected in parallel to avoid wasting hard-earned data from previous heavy channel recovery.

The IIFE can be calculated by the following equations:

(10) $$\begin{array}{cc} {\mathrm{CR}}_{i} & ={\mathrm{IRE}}_{i}\left({\mathrm{IF}}_{i-1}\right)\\ {\mathrm{SSD}}_{i} & =S({\mathrm{CR}}_{i})\\ {\mathrm{EF}}_{i} & ={\mathrm{IEE}}_{i}\left(upsampling\left(\mathrm{SD}-{\mathrm{SSD}}_{i}\right)\right)\\ {\mathrm{IF}}_{i} & ={\mathrm{ES}}_{i}\left({\mathrm{IF}}_{i-1},{\mathrm{EF}}_{i}\right) \end{array}$$

where ${\mathrm{IRE}}_{i}$ is realized by two 3×3 convolution and ReLU layers to extract a one-channel ${\mathrm{CR}}_{i}$. Then, ${\mathrm{SSD}}_{i}$ can be achieved by module S(·) defined in Equation (9). ${\mathrm{IEE}}_{i}$ also consists of two 3×3 convolution and ReLU layers to augment ${\mathrm{EI}}_{i}$ into ${\mathrm{EF}}_{i}$ that contains more helpful information. Then, ${\mathrm{IF}}_{i}$ is completely revised as a whole through ${\mathrm{ES}}_{i}$, by incorporating ${\mathrm{EF}}_{i}$ and ${\mathrm{IF}}_{i-1}$.

Therefore, it is believed that information flow in the main branch is protected and enhanced. To perfectly use the ${\mathrm{IF}}_{i}$ continuously produced by the previous network, IIFE shows great advantages in the resampling process of each iteration. It is emphasized that there are no steps of forced channel compression that will cause information loss. Under these circumstances, the method of effectively retaining and recovering more information can be found.

**Figure 8:**
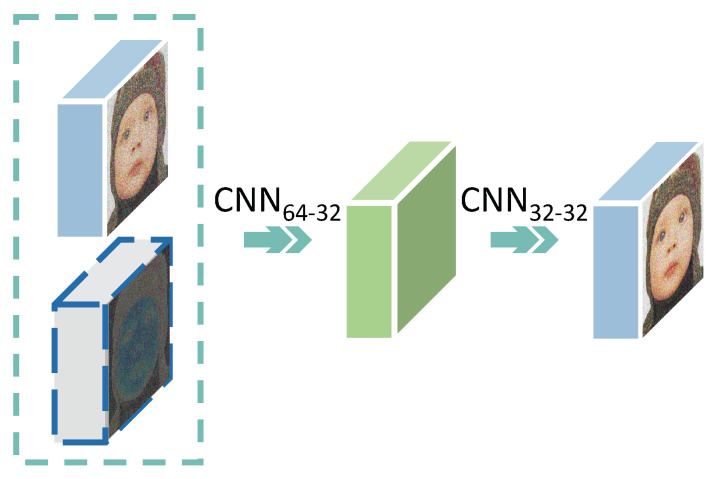
The fusion of errors in ES. At first, the two input feature maps ${\mathrm{IF}}_{i-1}$ and ${\mathrm{EF}}_{i}$, which are represented by blue and gray cubes, respectively, are concatenated. Then, the features are encoded into a hidden feature with 32 channels (shown as the green cube) and decoded to achieve the final result ${\mathrm{IF}}_{i}$. In this way, the full fusion between errors and intermediate features can be achieved. The encoder and decoder are realized by two 3×3 convolution and ReLU layers of $CNN_{64-32}$ and $CNN_{32-32}$, respectively.

In the meantime, a mechanism of error compensation is usually adopted through the simple pointwise addition of the two one-channel images, ${\mathrm{CR}}_{i}$ and ${\mathrm{EI}}_{i}$. It is believed that nonlinear mapping will be helpful for image reconstruction. Therefore, the ES module is designed and inspired by the encoder-decoder, as shown in Figure 8. Instead of simple addition, it is efficient to make the network learn which information to absorb and how to integrate due to the more adaptive and diversified fusion than addition. The performance of IIFE is far better than that shown in Figure 6 because the Φ, IRE, IEE, and ES modules are combined effectively, realizing outstanding information collection, transmission, supplementation, and fusion.

Finally, IIFE can cooperate with both ECCE and CIE to form a tight structure as IEF-CSNET. The upsampling module is replaced with CIE and ECCE to generate high-quality ${\mathrm{EEI}}_{i}$. For specific details of the combination, please refer to the overview of the pipeline in Figure 7 and the pseudocode in Algorithm 1 under Section 3.1.

## 4. Experiment

### 4.1. Settings

Datasets: The datasets are prepared for training and testing in the same way as the experimental details in [3]. COCO 2017 [37] is a large-scale dataset and is applied as the training set in this work by gathering images of complex everyday scenes containing common objects in their natural context. The patches with 128×128 pixels are cropped randomly without any data augmentation during the training. In addition, Set5 [38], Set11 [23], Set14 [39], BSD68 [40], and Urban100 [41] are employed as testing sets to evaluate the performance and robustness more comprehensively because they are widely applied in image reconstructions. Their specific information is listed in Table 1.

Training and Test Details: During the training, the batch size, $N_{I}$, and learning rate $L_{r}$ are set as 64, 12, and 0.0001, respectively. All images in the datasets are transferred into YCbCr format, and the luminance components (Y channel) are utilized for both training and testing, similar to what the reference papers performed. Because of the different resolutions, images in the test sets are processed with batch size = 1 one by one. Peak Signal-to-Noise Ratio (PSNR) and Structural Similarity (SSIM) [42] are employed to quantitatively evaluate the performance of the reconstructed images. The larger the PSNR and SSIM values are, the better the performance is [43].

All the implementations and experiments are deployed under the environment of open-source framework Pytorch 1.8.0 and CPU (Intel Xeon CPU E5-2678 v3 @ 2.50 GHz) with GPU (GeForce RTX 2080 Ti).

### 4.2. Quantitative Evaluation

The quantitative analysis results of all methods are provided in Table 2. PSNR and SSIM are tested on five testing sets and five different sampling rates (1%, 4%, 10%, 25%, and 50%) so that the effects of all methods can be objectively compared under different conditions. The results indicate that the proposed method achieves the best results under different sets or sampling rates. In addition, the smaller standard deviation than others reflects the higher robustness. As listed in Table 2, the average PSNR values of all testing sets are improved by 0.62, 0.6, 0.95, 1.32, and 0.99 dB under the five sampling rates. The absolute improvement under all sampling rates is helpful for practically applying the CS. Meanwhile, the inference speed is much higher than that of the SOTA methods [3] (see Section 4.4). The better reconstruction performance is attributed to the fact that the proposed method can match the characteristics of CS well and realize optimization avoiding the forced loss of intermediate results encountered by other methods. Meanwhile, it benefits from making full use of information in the compressed domain and intermediate results of previous and subsequent iterations to assist the reconstruction.

### 4.3. Qualitative Evaluation

**Figure 9:**
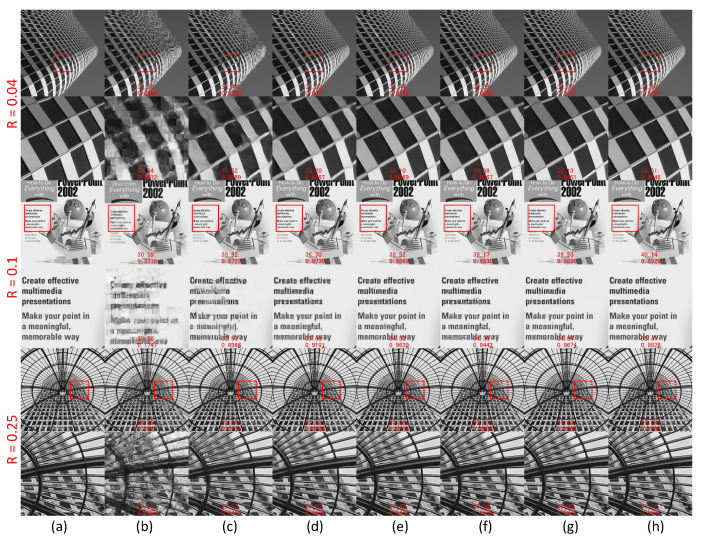
The results for qualitative evaluation. (**a**): Ground truth; (**b**): ReconNet; (**c**): ISTANet++; (**d**): CSNET+; (**e**): AMPNet; (**f**): COAST; (**g**): MADUN; (**h**): Proposed method. The images are randomly selected for comparison under the three sampling ratios of (0.04%, 0.10%, and 0.25%). The detailed parts of the whole image are marked with a red box and shown in an enlarged view below the corresponding image. The indicators of both the complete and enlarged images are calculated and listed.

For the qualitative evaluation, the performances of different methods can be compared based on the visual perception of the final output images. In Figure 9, three result sets (R=0.04%,0.10%, and 0.25%) are randomly selected to fully demonstrate the intuitive performance of reconstruction. The full images and the enlarged parts are displayed simultaneously to show the texture and edge more clearly. In addition, the PSNR and SSIM of the images and enlarged parts are both calculated and listed. The comparison shows that there are much fewer artifacts or blurred parts in the results from the proposed method in this work than that of the other counterparts. The comparison among different methods can fully prove that the proposed method shows greater advantages in processing texture details and high-quality images with vivid and sharp edges.

### 4.4. Inference Speed

The inference speed experiments are set as in reference [10] because detailed descriptions of the settings and results are provided. The number of images that can be processed by different methods per second are listed in Table 3, based on which the running speed can be compared more easily on the same hardware system. On the one hand, the inference speed of this work is obviously superior to that of the SOTA method [3] by orders of magnitude. On the other hand, the proposed method is slightly slower than the fastest method, Reconnet [23], but an additional BM3D denoiser must be cascaded after Reconnet, which will take more than 10 s for each 256 × 256 image in use and cannot be parallelized among images. Finally, it needs to be noted that the proposed method greatly improves the reconstruction performance compared with all other methods of approximate inference speed. The overall analysis results suggest that the proposed method realizes a stable and outstanding reconstruction and shows a speed advantage.

### 4.5. Ablation Experiment

For the ablation study, the effectiveness of the three designed submodules is explored and analyzed. To illustrate their improvement effects in CS reconstruction separately, four different configurations of IEF-CSNET are implemented, which are introduced as follows:

- W/O IIFE: No IIFE is set, but ECCE, CIE, and the base model in Figure 6 are a part of the network.
- W/O ECCE: No ECCE works, but the other two modules are employed.
- W/O CIE: No CIE is added, but the other two are considered.
- ALL: CIE, ECCE, and IIFE act with united strength.

The average PSNR and SSIM values of the five datasets under these settings are calculated. To evaluate the function of each module more comprehensively, two sampling rates, R=1% and 50%, are employed for testing, and the results are tabulated in Table 4. The IIFE module improves the performance most greatly, almost close to 1 dB higher than the base module in Figure 6. After supplementation with ECCE and CIE, the performance is still improved under both sampling ratios, even with the help of IIFE. In the case of an extremely low compression ratio, the absence of CIE (W/O CIE) will lead to a larger loss because the FESD obtained by CIE is critical in the reconstruction.

**Figure 10:**
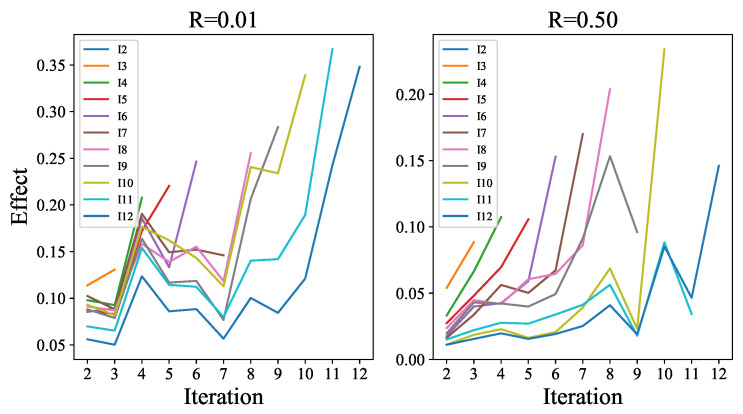
The internal interpretability of ECCE. Each line denoted as $I_{i}$ represents the weights of the convolution layer from ${\mathrm{ECCE}}_{i}$ in iteration *i*. On a specific polyline $I_{i}$, there are different weight responses for different input channels. Based on these weights, the importance of each channel in the whole task can be measured because the larger the weight is, the higher the proportion of information extracted from the corresponding input channel. Point (x,y) indicates how valuable the specific input channel (${\mathrm{EEI}}_{x},x\in \left[2,i-1\right]$ or ${\mathrm{CFEI}}_{i},x=i$) is in the calculation of ${\mathrm{ECCE}}_{i}$.

In addition, the average weights in the convolution layers of ECCE from different iterations are calculated to analyze the internal interpretability, as visualized in Figure 10. In the figure, the two sampling rates, R=1% and 50%, are employed to show the internal interpretability. On the one hand, all polylines $I_{i}$ from different ratios are basically in an upwards trend. This is because the closer the iteration is, the more contributive it will be to the current iteration results. The EEIs in previous iterations will guide the inference of ${\mathrm{EEI}}_{i}$ at this stage because their corresponding weights cannot be ignored by comparison. The network will be committed to recovering the problems not solved by previous iterations. However, the ${\mathrm{CFEI}}_{i}$ produced in the current iteration is decisive because the weight of this input channel is the largest.

Finally, all the submodules will be composed of the complete network structure of IEF-CSNET, and the best performance will be achieved under all compression ratios and different datasets.

## 5. Conclusions

In this work, a novel network architecture IEF-CSNET is proposed for high-performance image reconstruction based on CS. The IIFE to strengthen the information flow can enhance the efficiency of the whole recovery network and reduce the loss of information. The ECCE module, which closely connects the whole network, purposefully enhances the prediction of error images for higher performance in image restoration. The sensing module CIE allows the network to obtain a larger receptive field and can make full use of the information in the compressed domain. In this way, IEF-CSNET achieves the best reconstruction performance at this stage with the help of the above submodules and exhibits an improved operating speed by orders of magnitude from the SOTA method. Finally, these modules may be applied to other networks for image restoration networks and provide some reference for future work.

## Figures and Tables

**Table 1:** Summary of datasets.

Dataset	Number	Comments
Set5	5	Red-Green-Blue (RGB), unfixed resolutions
Set11	11	Gray, unfixed resolutions
Set14	14	2 Gray, 12 RGB, unfixed resolutions
BSD68	68	RGB, fixed resolution
Urban100	100	RGB, unfixed high-resolution city images

**Table 2:** The performances of different methods. All methods are tested under five testing datasets and five sampling ratios *R*. The indicators are shown in PSNR/SSIM format.

Methods	R	Set5	Set11	Set14	BSD68	Urban100	Avg ± Std
Reconnet [23]	1%	20.66/0.5211	19.34/0.4716	20.15/0.4650	21.16/0.4816	18.32/0.4261	19.92 ± 1.00/0.4731 ± 0.0305
	4%	24.45/0.6599	22.63/0.6115	23.16/0.5813	23.58/0.5760	20.82/0.5426	22.93 ± 1.21/0.5943 ± 0.0394
	10%	27.82/0.7824	25.87/0.7459	25.90/0.6937	25.79/0.6763	23.38/0.6697	25.75 ± 1.41/0.7136 ± 0.0436
	25%	31.93/0.8796	29.80/0.8578	29.28/0.8137	28.74/0.7965	26.84/0.8020	29.32 ± 1.64/0.8299 ± 0.0329
	50%	35.80/0.9350	33.89/0.9260	32.96/0.9013	32.22/0.8932	30.69/0.8954	33.11 ± 1.70/0.9102 ± 0.0170
	Avg.	28.13/0.7556	26.31/0.7225	26.29/0.6910	26.30/0.6847	24.01/0.6671	26.21 ± 1.31/0.7042 ± 0.0313
ISTA-Net++ [31]	1%	22.21/0.5872	20.43/0.5235	21.24/0.5118	22.09/0.5095	19.27/0.4682	21.05 ± 1.10/0.5200 ± 0.0384
	4%	26.53/0.7968	24.85/0.7528	24.79/0.6858	24.80/0.6557	22.71/0.6768	24.74 ± 1.21/0.7136 ± 0.0528
	10%	31.47/0.9111	29.82/0.8972	28.63/0.8220	27.64/0.7858	27.53/0.8513	29.02 ± 1.48/0.8535 ± 0.0465
	25%	36.09/0.9577	34.78/0.9569	33.03/0.9146	31.23/0.8939	32.48/0.9393	33.52 ± 1.72/0.9325 ± 0.0248
	50%	41.43/0.9824	40.19/0.9833	38.28/0.9672	36.08/0.9615	38.14/0.9794	38.82 ± 1.84/0.9747 ± 0.0088
	Avg.	31.55/0.8470	30.02/0.8227	29.19/0.7803	28.37/0.7613	28.03/0.7830	29.43 ± 1.26/0.7989 ± 0.0313
CSNET+ [26]	1%	24.57/0.6853	22.70/0.6257	23.20/0.6027	23.94/0.5876	21.03/0.5591	23.09 ± 1.21/0.6121 ± 0.0425
	4%	29.20/0.8799	26.78/0.8421	26.72/0.7816	26.58/0.7555	24.26/0.7658	26.71 ± 1.56/0.8050 ± 0.0480
	10%	32.97/0.9418	30.38/0.9188	29.68/0.8740	28.93/0.8519	27.26/0.8687	29.84 ± 1.88/0.8910 ± 0.0337
	25%	37.35/0.9721	35.00/0.9629	33.69/0.9407	32.55/0.9320	31.56/0.9423	34.03 ± 2.02/0.9500 ± 0.0150
	50%	42.47/0.9879	40.77/0.9876	38.75/0.9768	37.56/0.9772	36.96/0.9798	39.30 ± 2.05/0.9819 ± 0.0049
	Avg.	33.31/0.8934	31.13/0.8674	30.41/0.8352	29.91/0.8209	28.21/0.8232	30.59 ± 1.66/0.8480 ± 0.0281
AMPNet [4]	1%	24.74/0.6989	21.61/0.6201	23.41/0.6153	24.10/0.5967	21.34/0.5803	23.04 ± 1.35/0.6222 ± 0.0408
	4%	29.44/0.8878	26.13/0.8433	27.14/0.7884	26.82/0.7593	24.89/0.7842	26.88 ± 1.49/0.8126 ± 0.0465
	10%	33.84/0.9480	30.01/0.9202	30.43/0.8801	29.37/0.8551	28.67/0.8892	30.46 ± 1.79/0.8985 ± 0.0324
	25%	38.31/0.9750	35.12/0.9676	34.93/0.9470	33.20/0.9337	33.88/0.9566	35.09 ± 1.75/0.9560 ± 0.0147
	50%	43.53/0.9892	40.56/0.9868	40.08/0.9787	38.26/0.9774	39.34/0.9848	40.35 ± 1.77/0.9834 ± 0.0046
	Avg.	33.97/0.8998	30.68/0.8676	31.20/0.8419	30.35/0.8244	29.63/0.8390	31.17 ± 1.49/0.8545 ± 0.0266
COAST [44]	1%	24.05/0.6637	20.87/0.5836	22.70/0.5847	23.62/0.5749	20.74/0.5473	22.40 ± 1.37/0.5908 ± 0.0388
	4%	29.16/0.8813	25.55/0.8333	26.71/0.7816	26.56/0.7537	24.45/0.7738	26.49 ± 1.56/0.8048 ± 0.0464
	10%	33.36/0.9445	29.45/0.9159	29.99/0.8761	29.11/0.8517	28.06/0.8811	29.99 ± 1.80/0.8938 ± 0.0326
	25%	38.20/0.9742	35.03/0.9680	34.72/0.9465	33.08/0.9338	33.65/0.9565	34.94 ± 1.78/0.9558 ± 0.0145
	50%	42.81/0.9879	39.58/0.9857	39.13/0.9770	37.66/0.9760	37.96/0.9820	39.43 ± 1.83/0.9817 ± 0.0047
	Avg.	33.52/0.8903	30.10/0.8573	30.65/0.8332	30.00/0.8180	28.97/0.8281	30.65 ± 1.53/0.8454 ± 0.0259
MADUN [45]	1%	24.91/0.7161	21.80/0.6412	23.46/0.6269	24.17/0.6042	21.56/0.6044	23.18 ± 1.31/0.6386 ± 0.0412
	4%	29.94/0.8984	26.56/0.8595	27.41/0.7985	27.03/0.7682	25.56/0.8094	27.30 ± 1.46/0.8268 ± 0.0463
	10%	34.19/0.9503	30.42/0.9261	30.66/0.8856	29.59/0.8612	29.54/0.9052	30.88 ± 1.71/0.9057 ± 0.0310
	25%	38.82/0.9757	35.88/0.9714	35.42/0.9509	33.52/0.9378	34.85/0.9634	35.70 ± 1.75/0.9599 ± 0.0139
	50%	42.36/0.9862	39.31/0.9849	38.93/0.9746	36.99/0.9717	38.63/0.9839	39.25 ± 1.75/0.9802 ± 0.0059
	Avg.	34.04/0.9053	30.79/0.8766	31.18/0.8473	30.26/0.8286	30.03/0.8533	31.26 ± 1.45/0.8622 ± 0.0264
CSformer [3]	1%	25.22/0.7197	21.95/0.6241	23.88/0.6146	23.07/0.5591	21.94/0.5885	23.21 ± 1.24/0.6212 ± 0.0542
	4%	30.31/0.8686	26.93/0.8251	27.78/0.7581	25.91/0.7045	26.13/0.7803	27.41 ± 1.59/0.7873 ± 0.0562
	10%	34.20/0.9262	30.66/0.9027	30.85/0.8515	28.28/0.8078	29.61/0.8762	30.72 ± 1.97/0.8729 ± 0.0411
	25%	38.30/0.9619	35.46/0.9570	35.04/0.9316	31.91/0.9102	34.16/0.9470	34.97 ± 2.07/0.9415 ± 0.0188
	50%	43.55/0.9845	41.04/0.9831	40.41/0.9730	37.16/0.9714	39.46/0.9811	40.32 ± 2.08/0.9786 ± 0.0054
	Avg.	34.32/0.8922	31.21/0.8584	31.59/0.8258	29.27/0.7906	30.26/0.8346	31.33 ± 1.70/0.8403 ± 0.0339
IEF-CSNET	1%	25.26/0.7285	22.21/0.6533	23.88/0.6363	24.33/0.6090	22.04/0.6275	23.54 ± 1.24/0.6509 ± 0.0414
	4%	30.31/0.9016	26.98/0.8656	27.82/0.8033	27.17/0.7706	26.27/0.8247	27.71 ± 1.39/0.8332 ± 0.0461
	10%	34.64/0.9522	31.03/0.9324	31.09/0.8884	29.78/0.8626	30.29/0.9133	31.37 ± 1.71/0.9098 ± 0.0316
	25%	39.00/0.9758	36.20/0.9721	35.71/0.9519	33.65/0.9381	35.36/0.9656	35.99 ± 1.73/0.9607 ± 0.0139
	50%	44.17/0.9893	41.18/0.9877	40.65/0.9799	38.67/0.9791	40.29/0.9870	40.99 ± 1.80/0.9846 ± 0.0042
	Avg.	34.68/0.9095	31.52/0.8822	31.83/0.8519	30.72/0.8319	30.85/0.8636	31.92 ± 1.44/0.8678 ± 0.0265

**Table 3:** The number of images that can be processed by different methods per second. The inference of 256 × 256 images is executed 105 times, and the average running time of the next 100 times is taken as the final result of the running time *t* of each image. All images are processed with B=1. The number of pictures that can be processed per second is 1/t.

Methods	Ratio = 0.01	Ratio = 0.01
Reconnet	137.17	132.62
ISTA-Net++	44.80	44.84
CSNET+	93.02	91.32
AMPNet	39.95	37.52
COAST	24.76	24.87
MADUN	16.00	16.02
CSformer	-	0.20
IEF-CSNET	36.11	35.71

**Table 4:** The Ablation Experiment.

	R = 0.01		R = 0.5	
	PSNR	SSIM	PSNR	SSIM
W/O IIFE	23.40	0.6291	40.28	0.9833
W/O ECCE	23.77	0.6519	41.18	0.9848
W/O CIE	23.70	0.6479	41.24	0.9849
ALL	23.83	0.6551	41.31	0.9850

## Data Availability

Not applicable.
